# Mosquito (Diptera: Culicidae) larval ecology in natural habitats in the cold temperate Patagonia region of Argentina

**DOI:** 10.1186/s13071-019-3459-y

**Published:** 2019-05-07

**Authors:** Marta G. Grech, Luz M. Manzo, Luis B. Epele, Magdalena Laurito, Alfredo Ñ. Claverie, Francisco F. Ludueña-Almeida, María L. Miserendino, Walter R. Almirón

**Affiliations:** 1 Consejo Nacional de Investigaciones Científicas y Técnicas, CONICET, Centro de Investigación Esquel de Montaña y Estepa Patagónica (CIEMEP), Esquel, Chubut Argentina; 2 Universidad Nacional de la Patagonia San Juan Bosco, Facultad de Ciencias Naturales y Ciencias de la Salud, Sede Esquel, Esquel, Chubut Argentina; 3 Universidad Nacional de Córdoba, Facultad de Ciencias Exactas, Físicas y Naturales, Centro de Investigaciones Entomológicas de Córdoba, Córdoba, Argentina; 4 Consejo Nacional de Investigaciones Científicas y Técnicas, CONICET, Instituto de Investigaciones Biológicas y Tecnológicas (IIBYT), Córdoba, Argentina

**Keywords:** Pools, Physicochemical parameters, Models of probability of presence, Southern South America

## Abstract

**Background:**

Knowledge of immature habitats is an important focus for investigations of mosquito community ecology, and may improve our understanding of how environmental variables increase risk of mosquito-borne diseases by influencing the distributions and abundances of species. In Patagonia region, where climatic and ecological factors could be only borderline suitable for mosquito development, relatively little is known about larval ecology. The present study focuses on associations of environmental conditions in natural aquatic habitats with abundances of mosquito species that have colonized such habitats in Patagonia.

**Methods:**

We described the mosquito community composition within 26 natural temporary pools, and assessed the general relationships between environmental variables (pH, water temperature, conductivity, salinity, dissolved oxygen, aquatic plant cover and main nutrients) and larval abundances using redundancy analysis (RDA). Additionally, we compiled monthly climate data and vegetation indices for each larval habitat, and estimated the probability of presence for two of the most abundant species, describing through generalized linear models (GLM) the environmental, climatic and landscape variables-probability of occurrence relationships.

**Results:**

Seven species belonging to the genera *Culex* and *Aedes* were identified, with *Culex apicinus*, *Cx. acharistus* and *Aedes albifasciatus* being the most abundant. Mean larval densities were low (6.8 ± 2.8 larvae/dip), and the highest species richness and larval densities were recorded in northern and central areas. *Aedes albifasciatus*, a species of sanitary importance, was widely distributed, being the only one collected south of the 45th parallel of S latitude. RDA indicated that aquatic conductivity, pH, water depth, dissolved oxygen, ammonia and soluble reactive phosphorous accounted for the main part of the variation in the species composition. According to GLMs, wind speed was the variable that best described the presence of *Ae. albifasciatus*, and the probability of finding this species was positively associated with high wind speed values. On the other hand, the EVI vegetation index was the only variable included in the *Cx. apicinus* model, whereby there was a great probability of presence in arid areas with lower EVI values.

**Conclusions:**

Our results enhance our knowledge of larval habitat ecology under the extreme environmental conditions of Patagonia and will guide future efforts to understand how multiple effects can affect mosquito ecology and public health at higher latitudes.

**Electronic supplementary material:**

The online version of this article (10.1186/s13071-019-3459-y) contains supplementary material, which is available to authorized users.

## Background

Immature mosquitoes can develop in a wide range of aquatic habitats or breeding places where female mosquitoes lay eggs, larvae grow and pupate, and adults emerge [1]. The great diversity of immature habitats forms a gradient from small and highly ephemeral (e.g. bromeliad axils, tree holes, rock pools and human-made containers) to large and permanent (e.g. natural and artificial saline or fresh water bodies) [2]. These latter types of larval habitats commonly distributed in urban, rural and natural environments can include drains, canals, ditches, animal footprints, shallow wells, pools, retention ponds, swamps, marshes, stream and lake edges, and irrigated fields [3].

Organization of mosquito communities can be influenced by ecological context [2]. After female oviposition, the presence and abundance of immature stages are controlled by the environmental and physicochemical characteristics of the habitat (e.g. availability of food resources, microorganisms, pH, temperature, drying), and also by the occurrence of species interactions like intra or inter-specific competition, predation and mutualism [2, 4]. Moreover, ecosystem processes operating at different organization levels, and temporal and spatial scales, regulate the patterns of productivity of mosquito larval habitats in a larger landscape context [5, 6]. A growing body of literature indicates that one of the most important determinants for maintenance of adult populations is the presence and quality of immature breeding habitats, and may have implications for adult abundance, affecting their temporal and spatial distribution [6]. Previous studies evaluating natural and artificial habitats elucidate relationships between occurrences of species and larval habitat characteristics, identify some environmental and climatic variables that serve as drivers of vector larval abundance, identify areas with suitable habitats and evaluate the risk for disease transmission [4, 5, 7–13]. All this information is potentially useful for improving current control strategies, and should therefore contribute to more accurate predictions of the mosquito response to a changing environment [6].

There is literature available on mosquito larval habitats from several localities in northern and central areas of Argentina. These studies have mainly focused on artificial containers, permanent water bodies and temporary ground-level habitats. Additionally, a few studies refer to phytotelmata, gastrotelmata and rock pools as immature habitats [14]. In the cold-temperate Patagonia region, located in the southern cone of South America (36–55°S), a detailed understanding of mosquito larval ecology is still relatively lacking. The first mentions of mosquito geographical distributions in that region go back to 1927, where an entomological expedition to northwest Patagonia, on both the Argentine and Chilean sides, was performed. Larvae belonging to *Culex* and *Aedes* genera were collected in small pools next to rivers [15]. Currently, there is evidence of 16 mosquito species distributed in the Argentine Patagonia region belonging to the genera *Aedes*, *Culex* and *Orthopodomyia* [16], with Tierra del Fuego Province (52–55°S) being the southernmost area in the world where a mosquito species (*Aedes albifasciatus*) is permanently established [17].

The environmental heterogeneity in Patagonia is very important, since it could determine the ecological processes and patterns, and could also play an important role in shaping the distribution of mosquito species assemblages. *Aedes* and *Culex* species could be considered as widespread in this vast region, with *Ae*. *albifasciatus* being the most widely distributed [16]. Most knowledge of larval habitat characteristics relies on detailed descriptions of the breeding sites used by *Cx. eduardoi* [8] and *Ae*. *albifasciatus* [9]; however, little or no information is available on larval habitats for the remaining 14 species (Additional file 1: Table S1). Recent ecological and biological studies for *Ae. albifasciatus* populations from central Patagonia include egg thermal tolerance [18]; thermal effects on immature development and survival, and adult body size [19]; relationships between environmental variables and biting activity rate [20]; and wing-morphometrics [21].

In Argentina, arboviral diseases likely represent one of the major threats to public health, whereby mosquitoes belonging to the genera *Aedes* and *Culex* have been associated with *Flavivirus* dengue (DENV), yellow fever (YFV), St. Louis encephalitis (SLEV) and West Nile virus (WNV). They have also been incriminated in the transmission of *Alphavirus* western equine encephalitis (WEEV) and Venezuelan equine encephalitis (VEEV), and the *Orthbunyavirus* Cache Valley (CVV) and Kairi (KRIV) [22–24]. *Aedes albifasciatus* is a Neotropical mosquito widely distributed through Bolivia, Brazil, Uruguay, Chile and Argentina [17]. This floodwater species is considered one of the most bothersome mosquitoes, attacking humans and domestic mammals [25]. In Argentina, this species has been incriminated as potential vector of WEEV and CVV [26, 27]. During the WEEV epizootic in 1982–1983, recorded from northern and central areas of Argentina up to northern Patagonia (Río Negro Province); wild specimens of *Ae. albifasciatus* were found naturally infected by WEEV, and infection by this virus in horses and human were also notified [26]. Two CVV subtypes have also been isolated from *Ae. albifasciatus* mosquitoes [23]. Together with *Cx. pipiens*, both mosquito species have been incriminated as possible vectors of *Dirofilaria* species in Argentina [28].

Knowledge of mosquito larvae ecology in the southern Argentine Patagonia region remains restricted mainly to geographical distributions [16, 29] and some detailed habitat descriptions for two species [8, 9], but there has been no comprehensive study of the ecology of larvae under environmental conditions of this region, where climatic and ecological factors could be only borderline suitable for mosquito development. In this study, we test the general hypotheses: (i) Immature mosquitoes can develop in a wide range of natural habitats, under different ecological contexts and extreme environmental conditions of the Patagonia region; and (ii) Because environmental conditions in the Patagonia region are less suitable for mosquito proliferation toward the south (at higher latitudes), the spatial pattern of species richness varies across that region. Thus, the number of species will decrease from northern to southern areas.

Specifically, the present study focuses on associations of environmental conditions in aquatic habitats with abundances of mosquito species that colonized those habitats in Patagonia. Understanding the ecology of larvae is a central focus of mosquito community ecology and will be useful for developing control strategies in the region, where mosquitoes of sanitary importance like *Ae. albifasciatus* and *Cx. pipiens* are present, and arboviral activity was previously reported.

## Methods

### Study area

**Fig. 1:**
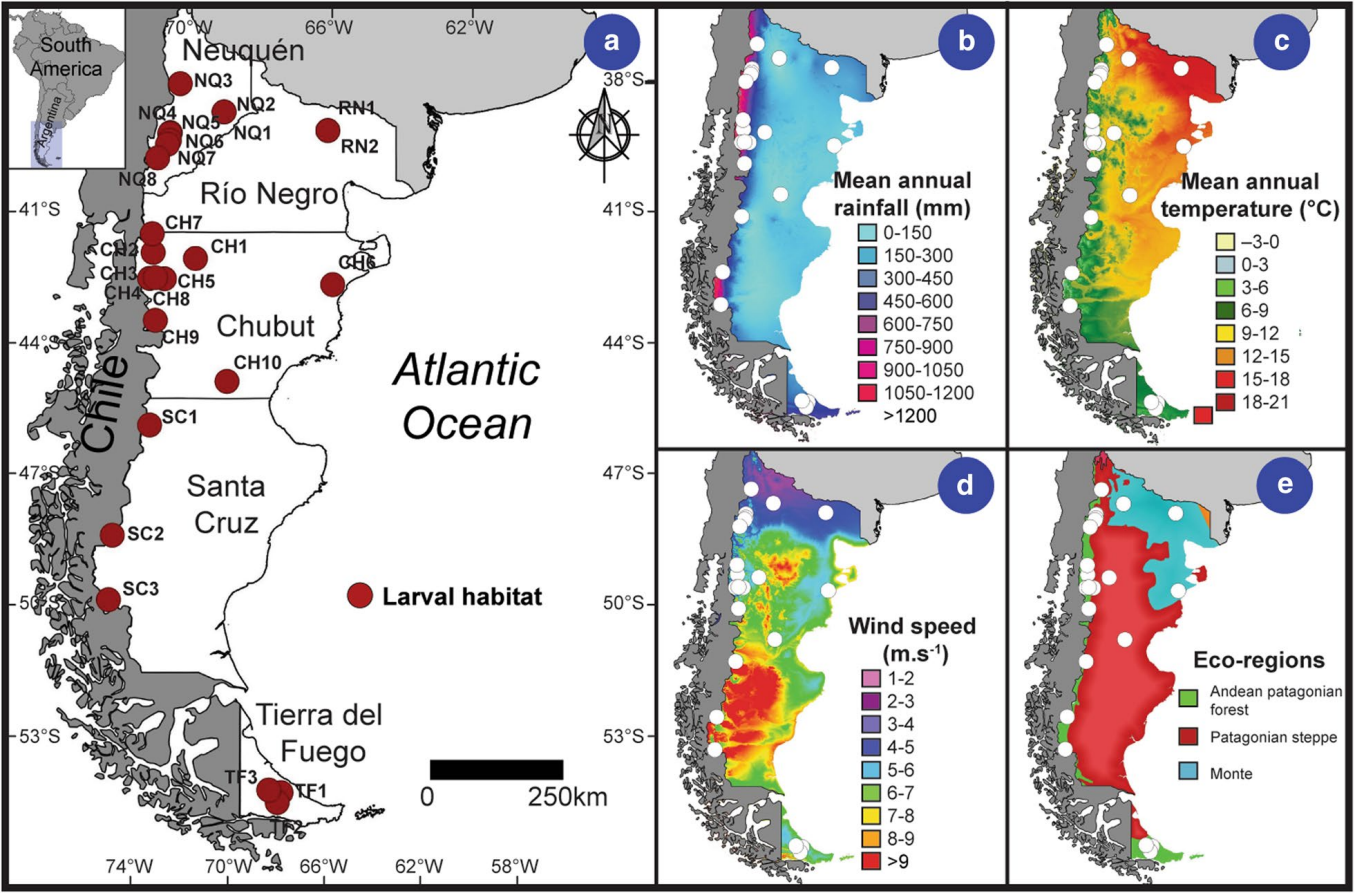
Study area. **a** Map of Patagonia region (Argentina) showing the locations of the 26 mosquito larval habitats surveyed in this study in Neuquén (NQ), Río Negro (RN), Chubut (CH), Santa Cruz (SC) and Tierra del Fuego (TF) provinces. **b** Mean annual rainfall. **c** Mean annual temperature. **d** Mean annual wind speed. **e** Ecoregions. Software: QGIS v.2.14 [30]. Interprovincial boundaries, climatic variables and ecoregions were derived from data produced by the IGN [31], WorldClim v.2 [32] and Burkart et al. [33], respectively

The Argentine Patagonia region, with an area of approximately 800,000 km^2^, is located in the southern extreme of South America, between 36–55°S, bordered to the east by the Atlantic Ocean and to the west by the Andean Mountains. From north to south, it extends about 1800 km, and comprises the provinces of Neuquén (NQ), Río Negro (RN), Chubut (CH), Santa Cruz (SC) and Tierra del Fuego (TF) (Fig. 1a). Patagonia can be defined as a temperate or cold-temperate region. The Andes impose an important barrier for humid air masses coming from the Pacific Ocean, resulting in a strong west-east gradient of precipitation across the Argentine side. Precipitation is mainly concentrated in winter months, and most of the central portion of Patagonia receives less than 200 mm per year [34] (Fig. 1b). Mean annual temperatures ranges from 12 °C in the northeastern part to 3 °C toward the south (Fig. 1c). Local factors such as topography and wind affect air temperature, and the strong westerly winds are characterized not only by their persistence during the year but also by their intensity [34] (Fig. 1d).

Based on geomorphology, hydrology, soils, vegetation and climate, Burkart et al. [33] described three ecoregions in Patagonia: (i) Monte; (ii) Patagonian steppe; and (iii) Andean Patagonian forest (Fig. 1e). The Argentinian Monte is an arid unit dominated by xeric shrublands. The climate is dry-temperate, with a mean annual temperature of 10–14 °C, receiving 100–200 mm of mean annual rainfall. The Patagonian steppe is the largest ecoregion in Patagonia. The climate is cold-arid, with a mean annual temperature decreasing from 10–14 °C in the north to 5–8 °C in the south, receiving less than 250 mm of mean annual precipitation. This unit is dominated by shrubs and herbs. The Andean Patagonian forest is mainly characterized by *Nothofagus* spp. The climate is cold-temperate and humid, with winter snows. Precipitation decreases following a west-east gradient, with 2000 mm in the Andean Mountains (near the Chilean side) and 700 mm toward its limit with the steppe. Mean annual temperatures also decrease from north (9.5 °C) to south (5.4 °C).

### Habitat characterization and mosquito sampling

The sampling of immature mosquitoes was performed in Patagonia region [38–54°S, 65–77°W, 14–1163 m above sea level (masl)], covering a total distance of 7000 km in two field trips. Twenty-six positive mosquito larval habitats were surveyed from December 2013 to January 2014 (Fig. 1a, Additional file 2: Table S2). Sampling sites were mostly distributed in western Argentine Patagonia, but they covered the main rainfall gradient. Habitats were located adjacent to the main paved routes and secondary unpaved roads, at varying distances (between 0–100 m from route edges). Each site was visited only once, and immature mosquitoes were sampled during daylight hours (between 9:00 h and 19:00 h). The first field sampling was conducted during December 2013 (late spring-early austral summer seasons) in NQ, RN and CH provinces. After that, during January 2014 (austral summer season) southern CH, SC and TF were surveyed in the second field trip.

The geographical coordinates and altitude of each site were measured with a handheld GPS (Garmin Etrex 10). We classified larval habitats as Monte, Steppe or Forest sites according to the ecoregions previously described. Natural water bodies were those not formed by anthropogenic actions and included pools, ponds, footprints and rock-pools. Only two of 26 habitats were artificial (e.g. ditch and pond). Habitats were mainly temporary pools or ponds. For temporary ponds (those with a hydroperiod between 6 and 12 months), the hydroperiod was estimated according to observations of maximum depth, water supply, and data collected from interviews of landowners. Turbidity was visually estimated, considering a sample turbid when the bottom of the white dipper used for collecting mosquito larvae could not be clearly seen. Each habitat was assigned to one of two sunlight exposure categories (sunny or shade) (Additional file 2: Table S2).

For each larval habitat, pH, water temperature (°C), conductivity (μS/cm), total dissolved solids (TDS) (mg/l), salinity (‰) and dissolved oxygen (DO) (mg/l) was measured *in situ* with a multiparameter probe (Hach sensION156; Hach, Loveland, U.S.). Using a field titration procedure, we determined alkalinity (meq/l). The air temperature (°C) was recorded with a digital thermometer. The percentage of aquatic plant cover was visually estimated and classified as indicated by [35]. Samples of water were removed from each site for measurements of soluble reactive phosphorous (SRP) (μg/l), nitrate + nitrite (NO_3_^−^ + NO_2_^−^) (μg/l), and ammonia (NH_4_^+^) (μg/l) (100 ml for SRP and NO_3_^−^ + NO_2_^−^; 200 ml for NH_4_^+^). Samples were field-filtered (cellulose acetate filter; Sartorius, Goettingen, Germany), taken back to the laboratory and stored frozen (−20 °C) until analyzed using standard methods [36–38], within 3 to 6 months. The average water depth (cm) was estimated from three measurements performed at random along the edges with a calibrated stick. Additionally, we measured length and width to estimate the larval habitat area (m^2^), using the following formula of an ellipse: Area_*i*_ = W_*i*_/2 × Π × L_*i*_/2, where, Area_*i*_ is the estimated area of the larval habitat _*i*_, W_*i*_ is the width of the habitat _*i*_, L_*i*_ is the length of the habitat _*i*_, and Π is PI a constant equal to 3.141592.

Additionally, we compiled monthly climate data for each larval habitat, extracted from WorldClim v.2 for the 1970–2000 period (spatial resolution *c*.1 km^2^) [32]. All data were aggregated to annual climate averages. We included mean annual temperature (°C), precipitation (mm), solar radiation (KJ m^−2^ day^−1^), wind speed (m/s), hydric balance (mm), minimum temperature of coldest month (°C), maximum temperature of warmest month (°C), precipitation of wettest (mm) and driest month (mm). We also used the normalized difference vegetation index (NDVI) and the enhanced vegetation index (EVI) to assess the vegetation canopy greenness at each site (a composite property of leaf area, chlorophyll and canopy structure) [39]. These two MODIS vegetation indices (16-day intervals; 250 × 250 m) were averaged to obtain a mean annual value, and aggregated across a target temporal range (February 2000 to January 2014).

Immature mosquitoes were collected using a 150-ml dipper. Seven samples were taken from each larval habitat to account for variation in microhabitat structure (e.g. aquatic vegetation and water column). In large sampling sites [area of 107 (NQ7) to 4398 m^2^ (SC2)], we took between 10 and 20 samples, trying to make the number of dips in each habitat proportional to the size of the habitat. Dipping took place where larvae usually breed, around the edges of water bodies, in shallow areas, and around aquatic vegetation. The density of immature mosquitoes was expressed as the number of larvae per dip (total number of larvae/number of dips). The collected material was transferred to small plastic flasks. The third- and fourth-instar larvae were killed and stored in 70% ethanol for taxonomic identification, according to available keys [40]. Young larvae (first and second instars) were reared to fourth instar for identification in small plastic flasks with water from the larval habitat. Whenever possible, fourth-instar larvae and pupae were individually reared to obtain larval and/or pupal exuviae and its associated adults. Specific identification was based on fourth-instar larvae or exuviae and male genitalia, according to the species, using descriptions and re-descriptions of the species and taxonomic keys [40]. We deposited the collected material in the mosquito collections of the Centro de Investigaciones Entomológicas de Córdoba (CIEC) (IIByT, CONICET-UNC) and CIEMEP (CONICET-UNPSJB).

### Data analysis

**Fig. 2:**
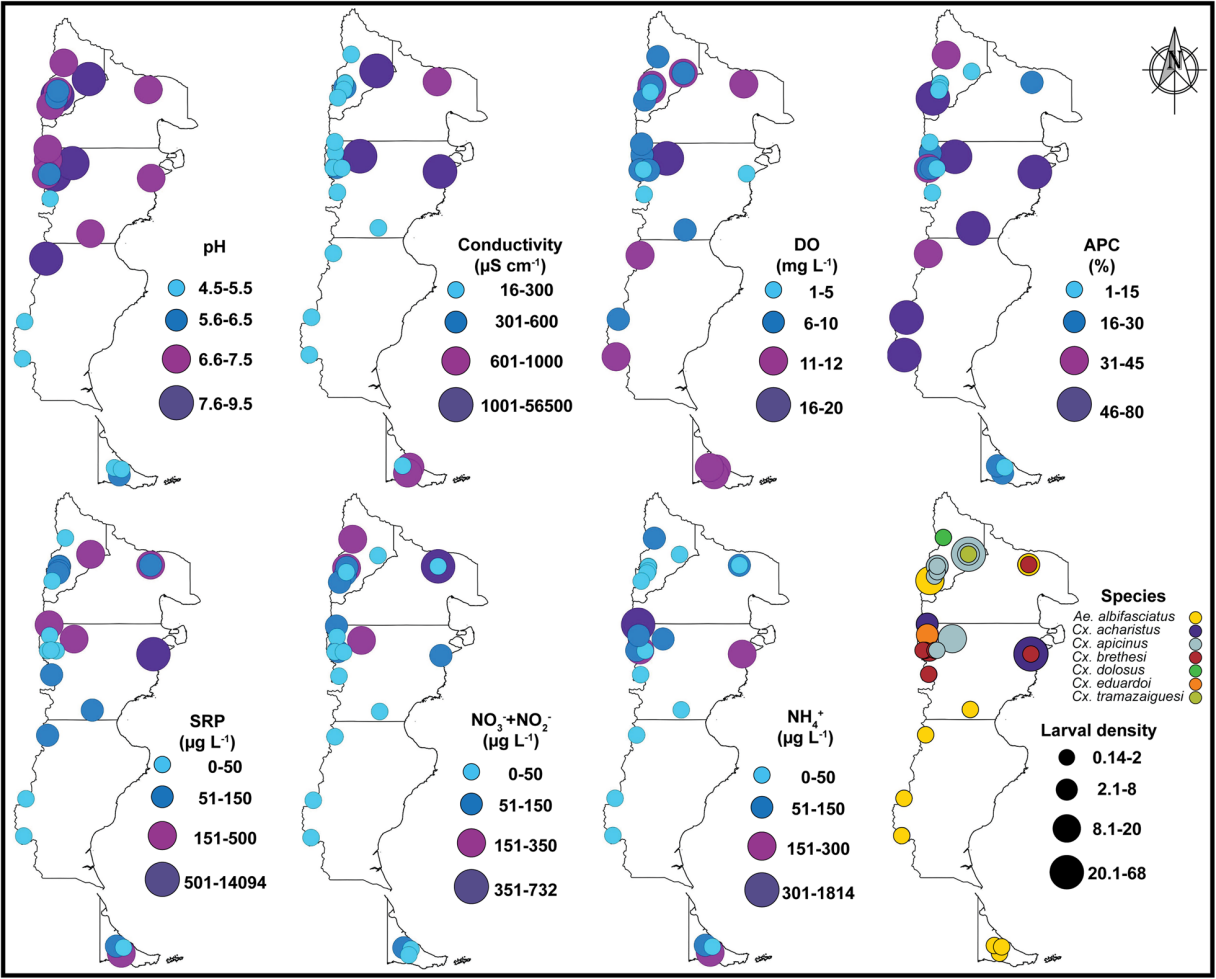
Spatial variation of mosquito species and environmental variables. Spatial variation of larval density, dissolved oxygen (DO), pH, conductivity, ammonia (NH_4_^+^), nitrate+nitrite (NO_3_^−^ + NO_2_^-^), soluble reactive phosphorous (PRS) and aquatic plant cover (APC) values measured in the 26 mosquito larval habitats. Software: QGIS v.2.14 [30]. Interprovincial boundaries were derived from data produced by the IGN [31]

We obtained descriptive summary measures in order to assess the variation ranges of media values of larval habitat characteristics and climatic variables. The cartographic work to generate Figs. 1 and 2 was performed using the open source software QGIS v.2.14 [30]. To estimate the extrapolated species richness in a species pool or the number of unobserved species, richness estimators were calculated. We used estimators based on incidences in sampling sites that give a single estimate for the collection of the 26 larval habitats (Chao, First order jackknife, Second order jackknife and Bootstrap), and estimators based on abundances on each site (Chao1). Additionally, species accumulation curves for the total of larval habitats were performed, using the species accumulation methods: collector (adds sites in the order they happen to be in de data); random (adds sites in random order); exact (finds the expected mean species richness); and coleman (finds the expected richness following Coleman et al. [41]). Statistical analyses were conducted using R software, v.3.2.4 [42] in RStudio software v.1.0.136 [43] and *vegan* package [44].

Associations of environmental variables in aquatic habitats with mosquito species abundance were evaluated using redundancy analysis (RDA). RDA is an ordination technique that considers species and environmental variables simultaneously and is particularly useful when there are underlying linear abundance distributions. Prior to determining the appropriate response model [linear (RDA) or unimodal (CCA)], a detrended correspondence analysis (DCA) was applied to our data. The length of the first DCA axis (which is scaled in units of standard deviation, SD) was <3 SD, indicating a homogeneous dataset for which linear methods like RDA are suitable [45]. Species data were log(x + 1) transformed, where x is the density of immature mosquito species. Environmental data were log transformed (except pH values) to improve normality. Site NQ6 was discarded from the analysis since measurements of NH_4_^+^ were not determined in that habitat. We performed the RDA analysis on the correlation matrix to avoid some variables becoming dominant just because of their large measurement units (e.g. conductivity). This standardization removes arbitrariness in the units of measurement of the environmental variables. To avoid collinearity between environmental variables, terms with variance inflation factors ≤5 were only allowed. Additionally, we looked at the pairwise correlations between variables, and removed variables with magnitudes greater than a pre-selected threshold of ± 0.5. Following this process caused three variables to be dropped from our analysis: TDS, salinity and alkalinity. Significance of the global model and the individual axes were tested by Monte-Carlo permutation test, with 999 permutations under the reduced model. Statistical analyses were carried out using R software v.3.2.4 [42], *vegan* [44] and *ggplot2* [46] packages, in RStudio software v.1.0.136 [43].

A by-species model approach was used to complement RDA analysis. Two separate generalized linear model analyses (GLM) were performed for two of the most abundant species, which also showed different distribution patterns: *Cx. apicinus* and *Ae. albifasciatus*. GLMs with a binomial family distribution and logit link function (this link constrains the predicted values to lie between 0 and 1) were run for each species, employing an automatic backward stepwise approach for model selection [47]. We tested models that included environmental variables measured in aquatic habitats, climate variables extracted from WorldClim v.2 and MODIS vegetation indices as fixed effects. The presence or absence of these species was the response variable. We checked collinearity between variables and removed variables with magnitudes greater than pre-selected thresholds (VIF = 5; r = ±0.5). To supplement parameter evidence of important effects, the model parameters were bootstrapped and confidence intervals limits (CL) of parameter estimates were calculated. Explanatory variables with CL including zero were excluded from the final model. To assess the classification accuracy of the selected model the Kappa index (K) was calculated, and the following ranges of agreement for the Kappa statistic were used: poor, K < 0.4; good, 0.4 < K < 0.75; and excellent, K > 0.75. This index indicates the model improvement over a random classification [48]. The cut-off point that provided the best value of K was also reported. Modelling was performed in R software v. 3.2.4 [42], *car* [49], *boot* [50] and *ggplot2* [46] packages, in the RStudio software v.1.0.136 [43].

## Results

### Characterization of mosquito larval habitats

Variation in the abiotic aquatic environment recorded at 26 larval habitats is shown in Table 1 and Fig. 2. The larval habitat area was highly variable ranging from 0.01 to *c.*4400 m^2^. Sites were mostly shallow with a maximum average water depth of 43 cm. Conductivity, TDS and salinity were highly variable across sites showing mean values of 4368.3 μS/cm, 2203.9 mg/l and 2.1‰, respectively. However, at more than 73% of the sites, the conductivity values were lower than 500 μS/cm. These three variables were negatively related to longitude and tended to show higher values to the east (mostly on Monte or Steppe sites) (Additional file 3: Table S3: *r*_conductivity_ = −0.6; *r*_TDS_ = −0.6; *r*_salinity_ = −0.5). The mean pH values varied between acidic (4.6) and alkaline (9.5) water and showed a negative relationship with latitude (Additional file 3: Table S3: *r* = −0.5). Additionally, sites varied from poorly to very well oxygenated, showing DO values between 1.3 and 19.3 mg/l. The percentage of aquatic plant cover ranged between 1–80% across sites, and the mean NO_3_^−^ + NO_2_^−^, NH_4_^+^ and SRP values were 77.5, 133 and 659.3 μg/l, respectively. This last variable was negative related to longitude, showing greater values toward the east (Additional file 3: Table S3: *r* = −0.5).

**Table 1:** Variation in the abiotic aquatic environment

Variable		Mean ± SE	Min	Max
Environmental variables	pH	6.8 ± 0.2	4.6	9.5
	Water temperature (°C)	21.4 ± 1.3	10.2	36.5
	Conductivity (μS/cm)	4368.3 ± 2845	16.5	56,500.5
	Total dissolved solid (mg/l)	2203.9 ± 1422.6	11.8	28,400
	Dissolved oxygen (mg/l)	8.9 ± 0.8	1.3	19.3
	Salinity (‰)	2.1 ± 1.4	0	25.1
	Alkalinity (meq/l)	4043.9 ± 1153.7	63.4	18,180
	Soluble reactive phosphorous (μg/l)	659.3 ± 538.1	0	14,090
	Nitrate + nitrite (μg/l)	77.5 ± 30.5	0	731
	Ammonia (μg/l)	133 ± 68.9	0.1	1813
	Water depth (cm)	15.7 ± 1.9	2.5	43.3
	Area (m^2^)	236.5 ± 176	0.01	4398.2
	Aquatic plant cover (%)	30.2 ± 5.3	1	80
	Air temperature *in-situ* (°C)	24.4 ± 0.07	10	37
Climatic variables	Hydric balance (mm)	-48.8 ± 72.5	-564	708
	Wind speed (m/s)	4.4 ± 0.2	2.8	6.5
	Temperature (°C)	9.7 ± 0.6	4.9	15.2
	Solar radiation (KJ m^−2^ day^−1^)	13,530 ± 464.8	9260	16,230
	Precipitation (mm)	622.4 ± 71.6	145	1324
	Min coldest month (°C)	-3.2 ± 0.2	-5.5	-0.7
	Max warmest month (°C)	21.4 ± 1	12.3	30.2
	Precipitation wettest month (mm)	94.6 ± 12.5	16	201
	Precipitation driest month (mm)	25.5 ± 3.1	8	55
Vegetation indices	NDVI	0.4 ± 0.03	0.2	0.7
	EVI	0.4 ± 0.02	0.2	0.7

*Notes*: Mean ± standard error (SE), and minimum and maximum values of environmental variables measured in the 26 mosquito larval habitats, climate variables extracted from WorldClim v.2 and MODIS vegetation indices. Measurements of ammonia were only carried out in 25 sites (*n*)

*Abbreviations*: Min, minimum; Max, maximum, SE, standard error

A total of 931 larvae were collected across Patagonia region, and seven mosquito species belonging to two genera were identified. *Culex apicinus* was the most abundant species (48.2%) restricted to northern Patagonia (NQ and CH). It was followed by *Cx. acharistus* (22.5%; CH) and *Ae. albifasciatus* (17.6%), the latter being the most widely distributed (NQ, RN, CH, SC and TF). Less abundant species were *Cx. brethesi* (7.7%; RN and CH), *Cx. eduardoi* (2.6%; CH), *Cx. dolosus* (1.4%; NQ), and *Cx.* (*Alm.*) *tramazaiguesi* (0.1%; NQ) (Fig. 2). The last species represented a new record for NQ province (det: M. Laurito) based on the male genitalia; voucher specimens are deposited in the mosquito collection of the CIEC.

In general, mean larval density values were mostly low, ranging between 0.14–68 larvae/dip. The highest species richness and larval densities were recorded in NQ (4 species) and CH (5 species) provinces. In SC and TF, only *Ae. albifasciatus* was present, showing low larval densities between 0.7–1.3 larvae/dip (Fig. 2). Associations between species pairs at the same larval habitat were not frequent (only at three sites). *Culex brethesi* with *Cx. acharistus*, and *Cx. brethesi* with *Cx. apicinus* were found together sharing the same breeding site in CH (CH6 and CH5). Furthermore, *Cx. apicinus* and *Cx. tramazaiguesi* were found coexisting in NQ1, in a habitat with the highest value of conductivity (56,500.5 μS/cm), TDS (28,400 mg/l), salinity (25.1‰), alkalinity (18180 meq/l), and basic pH value (8.4) (Fig. 2, Table 1).

The total richness estimators indicated between 8 (boot) and 11 (Chao and jack2) species for the 26 larval habitats, which is close to the observed 7 species. Estimations based on abundances on each site showed a good sample adequacy (Additional file 4: Table S4). Only for the collector method, the species accumulation curve reaches an asymptote after 15 sampling sites. The other three curves are not stable at the end, suggesting that mores sites are needed (Additional file 5: Figure S1).

### Associations of environmental variables with mosquito species abundance

**Fig. 3:**
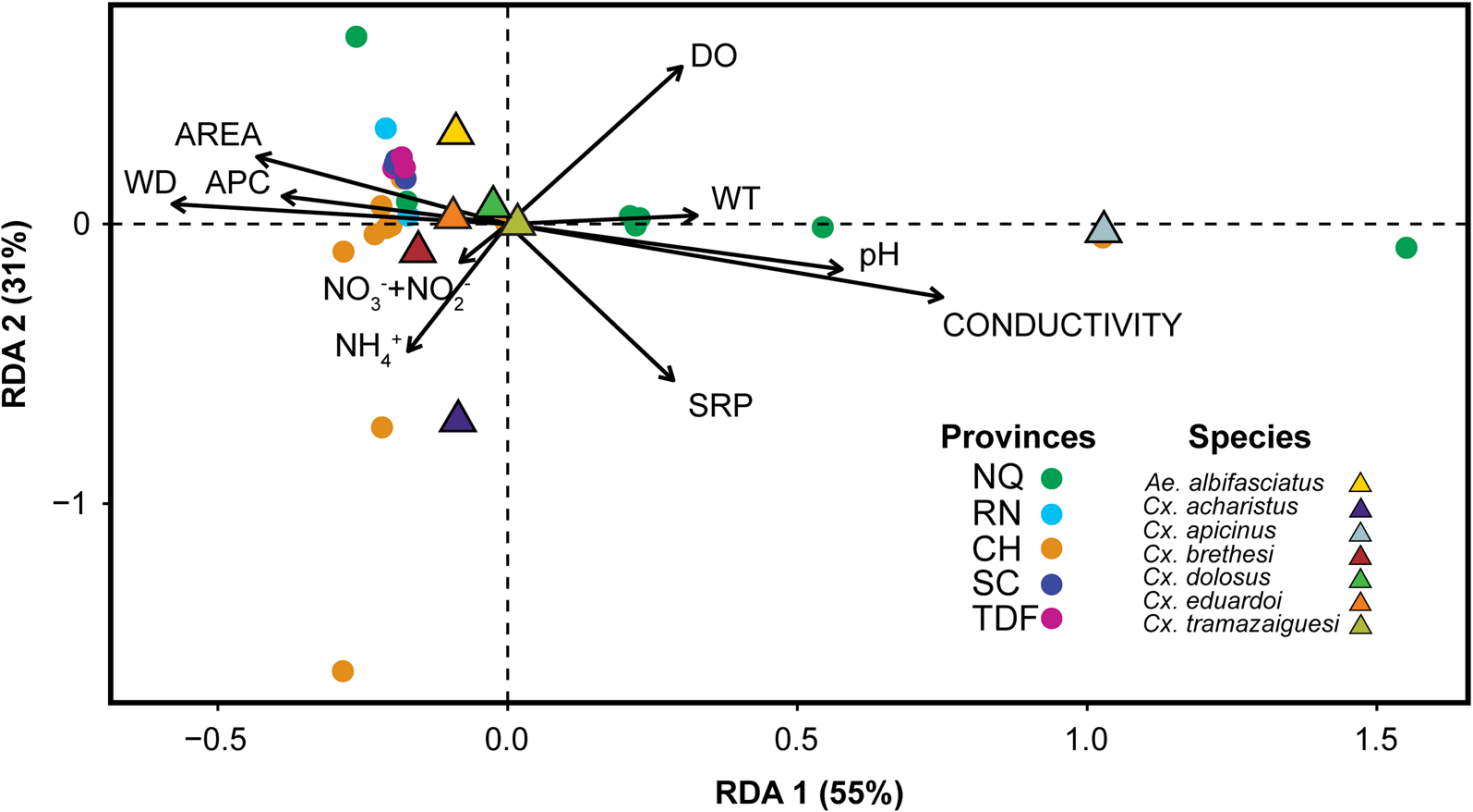
Redundancy analysis ordination diagram. RDA triplot with sites (circles), mosquito species (triangles), and environmental variables (arrows). The mosquito species are: *Cx. apicinus*, *Cx. acharistus*, *Cx. brethesi*, *Cx. eduardoi*, *Cx. dolosus*, *Cx. tramazaiguesi* and *Ae. albifasciatus*. The environmental variables are: dissolved oxygen (DO); water temperature (WT); pH; conductivity; soluble reactive phosphorous (PRS); ammonia (NH_4_^+^); nitrate+nitrite (NO_3_^-^+NO_2_^-^); water depth (WD); aquatic plant cover (APC); and area. Sites are shown in colored dots grouped into provinces: Neuquén (NQ); Río Negro (RN); Chubut (CH); Santa Cruz (SC); and Tierra del Fuego (TF)

An RDA-triplot of larval habitats, mosquito species and environmental variables based on the the first two axes explained 86% of the variance in the fitted species data (Table 2, Fig. 3). The first two axes and the global model were significant (Table 3). The first axis explained 55% of the variance and was defined by conductivity, pH and water depth. DO, together with NH_4_^+^ and SRP further differentiate on the second axis (31%) (Table 2, Fig. 3). The measured environmental variables relate strongly to the first two ordination axes, and they can account for the main part of the variation in the mosquito species composition. *Culex apicinus* was mainly found in habitats with the highest values of conductivity, pH and water temperature, represented on the bottom-right side of the diagram. *Culex acharistus*, in habitats with highest values of NH_4_^+^ on the bottom left-hand corner, and *Ae. albifasciatus* was more likely to be found in bigger and deeper sites, with the highest percentage of aquatic plant cover. The abundance of *Cx. brethesi* was positively associated with NO_3_^-^ + NO_2_^-^ (Fig. 3).

**Table 2:** Results of redundancy analysis

Redundancy analysis	RDA1	RDA2
Accumulated constrained eigenvalues:		
Eigenvalue	0.15	0.08
Proportion explained	0.55	0.31
Cumulative proportion	0.55	0.86
Loadings for constraining variables:		
pH	0.639	−0.179
Water temperature (°C)	0.362	0.034
Conductivity (μS/cm)	0.831	−0.29
Dissolved oxygen (mg/l)	0.333	0.624
Water depth (cm)	−0.641	0.079
Area (m^2^)	−0.479	0.267
Aquatic plant cover (%)	−0.43	0.111
Nitrate + Nitrite (μg/l)	−0.09	−0.15
Soluble reactive phosphorous (μg/l)	0.317	−0.619
Ammonia (μg/l)	−0.19	−0.504

*Notes*: RDA results for the first two axes showing the accumulated constrained eigenvalues and the loadings for the constraining variables

**Table 3:** Results of Monte-Carlo permutation test

Axis	*df*	F value	*P-*value
**RDA1**	**1**	**15**	**0.001**
**RDA2**	**1**	**8.4**	**0.001**
RDA3	1	2.5	0.061
RDA4	1	1.04	0.4
RDA5	1	0.5	0.8
RDA6	1	0.04	1
RDA7	1	0.003	1
**Global test**	**10**	**2.3**	**0.004**

*Notes*: Axis from the redundancy analysis, and degrees of freedom (*df*), F statistics and *P*-values derived from Monte-Carlo permutation test. Significant effects are highlighted in bold

### Models for mosquito species presence

**Fig. 4:**
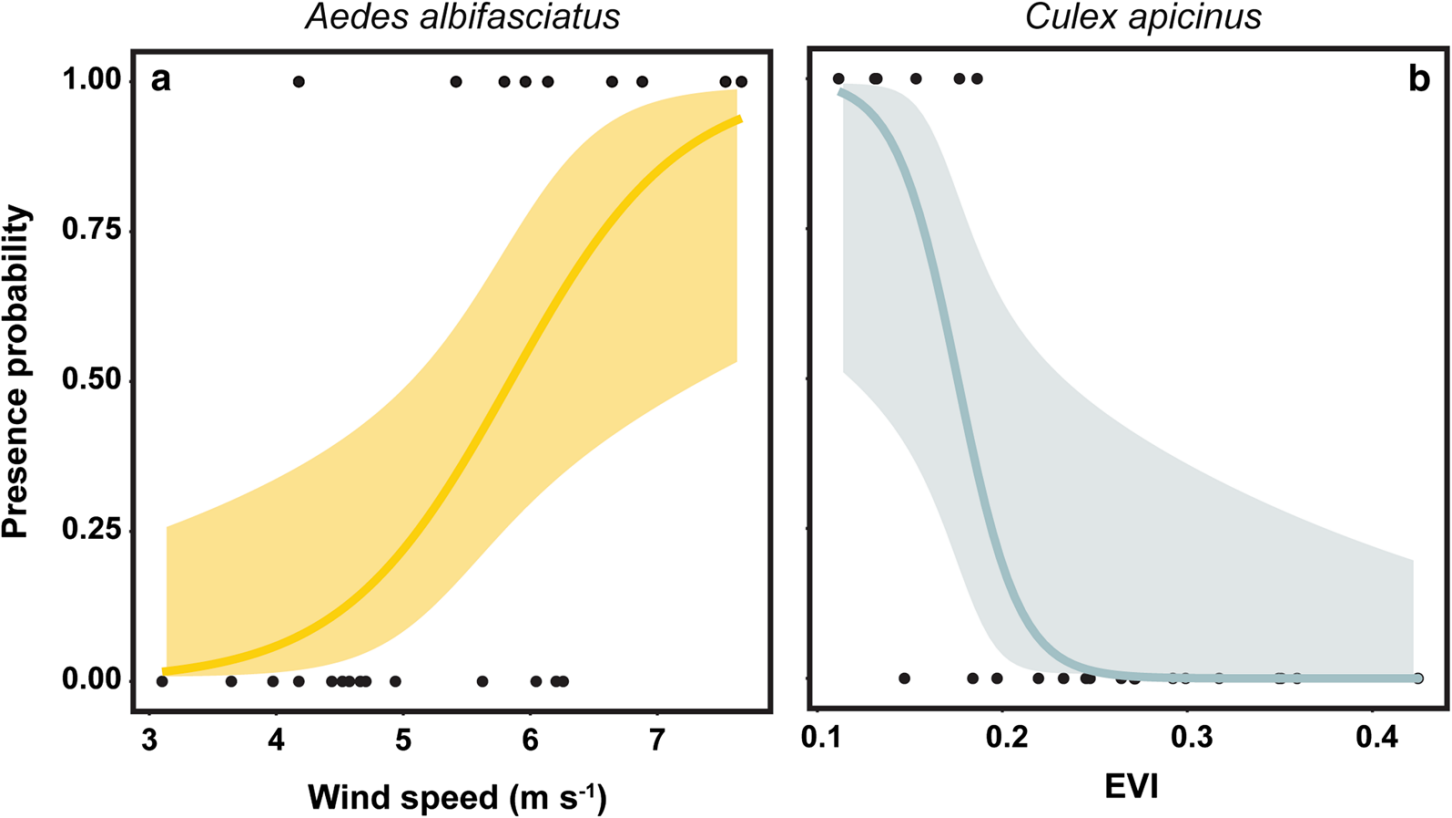
Probability of *Aedes albifasciatus* and *Culex apicinus* presence. Fitted values (solid line) obtained by the binomial generalized linear models with associated 95% confidence interval (shaded area). The black dots are the observed values. **a** Probability of presence of *Aedes albifasciatus* as a function of wind speed. **b** Probability of presence of *Culex apicinus* as a function of EVI

The best-fitting models of probability of presence differed among mosquito species (Table 4). For *Ae. albifasciatus*, the best model included only wind speed. Model parameter estimates and confidence intervals indicated a positive effect of this variable on the probability of presence of *Ae. albifasciatus* (Table 4, Fig. 4a). The mean wind speed was greater in sites (*n* = 9) with presence of this species (6.2 ± 0.4 m/s), and differed from sites (*n* = 17) without *Ae. albifasciatus* (4.5 ± 0.2 m/s). For *Cx. apicinus*, the best model included only the EVI vegetation index (negative effect), with a greater probability of presence in sites with lower values of EVI (Table 4, Fig. 4b). The mean EVI value was lower in sites (*n* = 8) with presence of *Cx. apicinus* (0.15 ± 0.01), compared to those without this species (0.3 ± 0.01) (*n* = 18).

**Table 4:** Generalized linear models results

Model	Explanatory variables	β ± SE	*z*-value	*P-*value	Lower CL	Upper CL	K	Cut-off point
*Aedes albifasciatus*	Intercept	−8.8 ± 3.4	−2.6	0.009	−21	−3	0.6	0.21
	Wind speed	1.5 ± 0.6	2.5	0.01	0.5	3.7		
*Culex apicinus*	Intercept	10.5 ± 4.9	2.1	0.03	2.2	275.2	0.82	0.22
	EVI	−59.9 ± 27.6	−2.2	0.03	−1388.9	−17.6		

*Notes*: GLMs results for the effect of environmental and climate variables, and vegetation indices on *Aedes albifasciatus* and *Culex apicinus* presence or absence. Explanatory variables, parameter estimates (β) (**± **standard error, SE), confidence limits (CL), kappa (K) statistic and cut-off points values are shown

## Discussion

The present study describes mosquito larvae community composition within natural temporary pools and establishes general relationships between environmental variables in aquatic habitats and larval abundances. Seven mosquito species belonging to the genera *Culex* and *Aedes* were identified across Patagonia region, with the highest species richness and larval densities recorded in northern and central areas between 38–43°S latitude. Rossi [16] updated a list of mosquito species present in Argentina and described a similar pattern of species richness in Patagonia, with the highest number of species found in northern areas. However, the total number of species recorded by provinces, and across the region, was higher than the present study with a total of 16 species belonging to the genera *Aedes*, *Culex* and *Orthopodomyia* (Additional file 1: Table S1). Surveying low diversity assemblages (like those with relatively low species richness and low abundances) is particularly hard, increasing the difficulty for recording new specimens [51, 52]. The lower observed richness in this study may in part be explained by some rare species with low population densities that will not be collected by chance, or small populations that are more likely to go extinct locally due to random fluctuations [53]. Additionally, we mostly focused on mosquito larvae breeding in natural temporary pools and did not survey a wider range of aquatic habitats (e.g. human-made containers, tree holes, stream and lake edges).

*Culex apicinus*, *Cx. acharistus* and *Ae. albifasciatus* were the most abundant species and showed different distribution patterns across the studied region. The first two species were present between 38–43°S latitude, whereas *Ae. albifasciatus* was widely distributed, being the only species collected south of the 45th parallel of S latitude at which minimum mean temperature of the coldest month reaches −3.4 °C. *Culex apicinus* and *Cx. acharistus* are widely distributed through northern and central Argentina, up to SC and CH provinces, respectively [16, 17]. The sanitary importance of both species is yet unknown. However, *Cx. apicinus* was found naturally infected with SLEV genotype V in central Argentina [54]. *Aedes albifasciatus* has been described previously as one of the most common species in Patagonia [29], and currently is the only species present in the southernmost area [16], and in the neighboring Magallanes region of Chile (51–53°S, 70–73°W) [55]. This species of sanitary importance is widely regarded as extremely tolerant of a range of temperatures within its distribution area in southern South America, and has been found to be adapted to the extreme environmental conditions of Patagonia [18, 19].

The less abundant species *Cx. brethesi* was widely distributed in northern and central Patagonia, but in low abundance, whereas, *Cx. eduardoi*, *Cx. dolosus* and *Cx. tramazaiguesi* remain as isolated records, the last of which is a new record in NQ province. *Culex tramazaiguesi* is a single species of the subgenus *Allimanta*, only known in Argentina [56]. Its epidemiological importance is yet unknown. It was previously found in subtropical and temperate regions of the country, with RN province as its southernmost limit of distribution [16]. In our study, *Cx. tramazaiguesi* showed the ability to proliferate under extreme environmental conditions in a habitat with the highest values of conductivity, TDS, salinity and alkalinity. Although most mosquitoes are restricted to freshwater, a number of species can develop in extremely high concentrations of salts [57]. Similar results were observed for *Cx. tramazaiguesi* [56, 58, 59], where larvae were found in hypersaline temporary pools, with similar mean pH (9.3) and alkalinity (16.6044 meq/l) values.

The presence of immature stages in a breeding site is usually the result of female oviposition choice and the quality of those habitats [4]. We determined that aquatic conductivity, pH, water depth, DO, NH_4_^+^ and SRP accounted for the main part of the variation in the mosquito species composition (RDA ordination). Many studies have used most of these variables for the assessment of mosquito larval habitats, but results have been inconsistent. The effect of these variables on mosquito larval abundance have shown different responses, and the nature of this variation seems to be context-specific. In general, larvae of most mosquito species can tolerate acidic or alkaline conditions in nature with pH values between 3–11 [1]. Positive associations were observed between *Culex* species larval abundance and pH, with pH values ranging between 6.8–8.5 [10], 6.4–8.2 [60] and 7.3–11.4 [61]. However, other authors reported negative associations [62], or no relationship between this variable and immature *Culex* [7, 8, 11]. Conductivity and DO are two of the most useful and commonly measured water quality parameters. The relevance of these variables on mosquito larval abundance has been shown for *Culex* species, with some mixed results [60, 63]. Although mosquito larvae primarily consume atmospheric oxygen, some species have also the ability to use DO [1, 64]. Previous studies indicated that mosquito larvae are unaffected by DO depletion; however, under experimental conditions, reduced levels of DO resulted in reduced larval survival and prolonged development time for *Cx. pipiens* [64]. Furthermore, certain solutes have been described in the literature as harmful to the larvae. High levels of NH_4_^+^, NO_3_^−^ and NO_2_^−^ can be toxic to some species of mosquitoes [57], but the effect of these nutrients on larval abundance are unclear [65]. According to Noori et al. [66], the type and amount of nutrients available in aquatic habitats produce differential effects on immature development for *Cx. quinquefasciatus*.

A growing body of literature on mosquito larval habitats has indicated that larvae of *Cx. apicinus* seem to be restricted to human-made containers [11, 67–70]. However, we found this species mainly in natural temporary pools associated with higher values of conductivity, water temperature and pH. In agreement, *Cx. apicinus* was collected less frequently from natural breeding sites including pools, spring and creek tributaries [7, 29]. In this study, *Cx. acharistus* showed a positive association with NH_4_^+^. Few studies have documented the immature habitats of this species, collecting larvae from artificial [11, 68, 69], natural [11, 29], and also phytotelmata habitats [71]. *Aedes albifasciatus* usually displays a wide diversity of habitats, being found mainly in temporary freshwater bodies [9, 11, 12, 25]. We found *Ae. albifasciatus* in bigger and deeper sites, with the highest percentage of aquatic plant cover. These results are coincident with those reported by Fischer et al. [12], where the proportion of habitats positive for this species was positively related to pool surface, depth, duration and vegetation cover. A positive relationship between larval abundance of *Ae. albifasciatus* and percentage of grasses was also observed south of the 53rd parallel of S° latitude [9]. It seems that vegetation can act as a source of nutrients that fuel microbial growth providing food for developing mosquito larvae, providing protection to the eggs from extreme temperatures, and increasing the availability of shelter from potential predators [9, 12, 72].

In our study, values of environmental variables measured in aquatic habitats, climate variables and vegetation indices varied across the studied region, and according to the best-fitting models these variables have differential effects on the probability of presence of mosquito species. Only climatic (wind speed) and landscape variables (vegetation index) were retained in the final models. Wind speed was the variable that best described the distribution of *Ae. albifasciatus*, and the probability of finding this species was positively associated with high wind speed values. Mosquitoes usually disperse, find mates, lay eggs and seek hosts in flight, but the flight of adults can be depressed by wind speeds greater than 3 km/h (*c.*0.8 m/s) [73]. Studies on *Ae. albifasciatus* have documented a reduced mosquito capture rate with increased wind speed [20, 74, 75], as well the fact that this species could be transported by wind [76]. Additionally, it was suggested that female *Ae. albifasciatus* could exploit periods between bursts of up to 35 km/h [74] and 55.5 km/h [20] to fly to their hosts. These results suggest that this species may be adapted to windy conditions. However, since in the present study *Ae. albifasciatus* was the only species present in those habitats from southern Patagonia, this may lead to biases in the estimated parameter. For that reason, future studies examining that relationship and the potential behavioral strategies involved are needed. The EVI vegetation index was the only variable included for the *Cx. apicinus* model. Many studies have highlighted the potential key role of satellite data in ecology of insects and vertebrates, and in particular the NDVI, this last variable being a widely available vegetation index with pre-processed data at various spatial scales [77]. Relationships between vegetation indices and *Culex* mosquito abundance have been previously described [78], and the NDVI has been also applied in mosquito studies as a surrogate measure of humidity and precipitation [79]. In this study, the probability of presence of *Cx. apicinus* was greater in sites with lower values of the vegetation index. These arid areas with lower EVI values, as a result of low or relative sparse plant cover, correspond to the Monte and Patagonian steppe regions and seem to be more suitable habitats for *Cx. apicinus*.

## Conclusions

The present study, performed at regional scale level, enhances our knowledge of mosquito larval habitat ecology under the environmental conditions of Patagonia. Spatial patterns of mosquito species distribution and their relationships with environmental variables were synthetized, including the presence of *Ae. albifasciatus*, a species of sanitary importance that is widely distributed through South America. Field studies and laboratory experiments frequently tend to focus on the geographical areas where mosquito-borne diseases are most severe, and where vector species are most abundant. However, in recent decades, the expansion of the geographical ranges of vector species into new areas, potentially driven by environmental change, has been documented [80]. In this sense, understanding the larval ecology in areas like Patagonia, where environmental conditions could be only borderline suitable for mosquito development and survival, will be potentially useful for developing models of population dynamics, and improving current mosquito control strategies. Additionally, future studies within a hypothesis-driven research framework should experimentally test for the effects of interspecific interactions (e.g. competition, predation) on mosquito larval communities, under different ecological contexts from Patagonia (e.g. size of habitats, hydroperiod, presence and coverage of aquatic plant, land use and main nutrients content, among others). These could help us to better understand how multiple effects can affect mosquito ecology and public health at higher latitudes.

## Additional files

**Additional file 1: Table S1.** Mosquito species present in Patagonia region. Literature review about the mosquito species present in Patagonia region (Argentina) and their larval habitat descriptions. *Abbreviation*: NDA, no data available.

**Additional file 2: Table S2.** Description of mosquito larval habitats employed in this study. Geographical coordinates, altitude (masl) and ecoregions are provided. Patagonian provinces: Neuquén (NQ), Río Negro (RN), Chubut (CH), Santa Cruz (SC) and Tierra del Fuego (TF).

**Additional file 3: Table S3.** Spearman rank correlations matrix. Spearman correlations between all environmental variables measured in the 26 mosquito larval habitats, climate variables extracted from WorldClim v.2 and MODIS vegetation indices. Variables: pH; WT, water temperature; conductivity; TDS, total dissolved solid; DO, dissolved oxygen; salinity; alkalinity; APC, aquatic plant cover; PRS, soluble reactive phosphorous; NO_3_^-^+NO_2_^+^, nitrate+nitrite; NH_4_^+^, ammonia; WD, water depth; area; Air T, *in situ* air temperature; Hydric B, hydric balance; Wind S, wind speed; temperature; Solar R, solar radiation; precipitation; Min CM, minimum temperature of coldest month; Max WM, maximum temperature of warmest month; Pre WM, precipitation of wettest month; Pre DM, precipitation of driest month; NDVI; EVI; latitude and longitude. Correlation coefficients higher than 0.5 are highlighted in bold.

**Additional file 4: Table S4.** Species richness estimators. Species richness estimators (± standard error) and number of observed species for the total of the 26 mosquito larval habitats, and for each site. Chao; Jack1: first order jackknife; Jack2: second order jackknife and Boot: bootstrap.

**Additional file 5: Figure S1.**Species accumulation curves. Species accumulation curves for the 26 mosquito larval habitats, using the species accumulation methods: collector (**a**), random (**b**), exact (**c**) and coleman (**d**).

## Abbreviations

NQ: Neuquén Province
RN: Río Negro Province
CH: Chubut Province
SC: Santa Cruz Province
TF: Tierra del Fuego Province
WT: water temperature
TDS: total dissolved solid
DO: dissolved oxygen
APC: aquatic plant cover
PRS: soluble reactive phosphorus
NO_3_^-^+NO_2_^+^: nitrate + nitrite
NH_4_^+^: ammonia
NDVI: normalized difference vegetation index
EVI: enhanced vegetation index
WD: water depth
Air T: *in situ* air temperature
Hydric B: hydric balance
Wind S: wind speed
Solar R: solar radiation
Min CM: minimum temperature of coldest month
Max WM: maximum temperature of warmest month
Pre WM: precipitation of wettest month
Pre DM: precipitation of driest month
RDA: redundancy analysis
GLM: generalized linear models
