# Supplementary material for: Mosquito (Diptera: Culicidae) larval ecology in natural habitats in the cold temperate Patagonia region of Argentina
Source: Parasit Vectors. 2019 May 7;12:214. doi: 10.1186/s13071-019-3459-y (PMC6505294; doi:10.1186/s13071-019-3459-y)
Supplement: Supplementary file 1 — Additional file 1: Table S1. Mosquito species present in Patagonia region. Literature review about the mosquito species present in Patagonia region (Argentina) and their larval habitat descriptions. Abbreviation: NDA, no data available. [file 13071_2019_3459_MOESM1_ESM.docx]

**Additional file 1: Supplementary Table 1.**

|  | **Argentine Patagonian Provinces** | | | | | | |  | |  |
| --- | --- | --- | --- | --- | --- | --- | --- | --- | --- | --- |
| **Species** | | **Neuquén** | **Río Negro** | **Chubut** | **Santa Cruz** | **Tierra del Fuego** | **Breeding site** | | **Reference** | |
| *Aedes (Ochlerotatus) albifasciatus* | | X | X | X | X | X | Roadside pools with grasses and reduced water turbidity, irrigation ditch, river bank, swamp formed by the overflowing of a river, farm-lands with irrigation systems | | Burroni et al. 2003, 2013; Marinone 2002; Rossi et al. 2011; Rossi 2015 | |
| *Aedes (Ochlerotatus) scapularis* | |  | X |  |  |  | NDA | | Rossi 2015 | |
| *Aedes (Ochlerotatus) serratus* | |  | X |  |  |  | NDA | | Rossi 2015 | |
| *Aedes (Stegomyia) aegypti* | | X |  |  |  |  | NDA | | Rossi 2015 | |
| *Culex (Allimanta) tramazaiguesi* | |  | X |  |  |  | NDA | | Rossi 2015 | |
| *Culex (Culex) acharistus* | | X | X | X |  |  | Creek tributary, spring tributary | | Muzón et al. 2010; Rossi et al. 2011; Rossi 2015 | |
| *Culex (Culex) apicinus* | | X | X | X | X |  | Creek and river tributary, spring creek | | Muzón et al. 2005; Rossi et al. 2008, 2011; Rossi 2015 | |
| *Culex (Culex) articularis* | | X | X | X |  |  | Creek tributary, stream with abundant riparian vegetation (*Cortadeira*) and associated with amphipods (*Hyalella*), spring creek with abundant riverside vegetation | | Muzón et al. 2010; Laurito et al. 2011; Rossi et al. 2011; Rossi 2015 | |
| *Culex (Culex) brethesi* | |  | X | X |  |  | NDA | | Rossi 2015 | |
| *Culex (Culex) coronator* | |  | X |  |  |  | NDA | | Rossi 2015 | |
| *Culex (Culex) dolosus* | | X | X | X |  |  | NDA | | Rossi 2015 | |
| C*ulex (Culex) eduardoi* | | X |  | X | X |  | Semi-permanent water bodies with rooted and free-floating aquatic plants, road ditch close to a river, stream tributary, natural water bodies at ground level | | Burroni et al. 2007; Grech et al. 2012; Rossi et al. 2011; Rossi 2015 | |
| *Culex (Culex) pipiens* | | X | X | X | X |  | Creek and river tributaries, irrigation ditch | | Rossi et al. 2011; Rossi 2015 | |
| *Culex (Culex) spinosus* | |  | X |  |  |  | NDA | | Rossi 2015 | |
| *Culex (Phytotelmatomyia) castroi* | |  | X |  |  |  | NDA | | Rossi 2015 | |
| *Orthopodomyia peytoni* | |  | X |  |  |  | NDA | | Rossi 2015 | |
| TOTAL SPECIES: 16 | | 8 | 14 | 8 | 4 | 1 | - | | - | |

**References**

Burroni N, Loetti V, Freire G, Marinone C, Schweigmann N, Jensen O. Characterization of larval habitat *Ochlerotatus albifasciatus* (Diptera: Culicidae) in Patagonia Argentina. Bol Soc Zool Urug. 2003;1:140.

Burroni N, Loetti V, Freire G, Jensen O, Schweigmann N. New record and larval habitats of *Culex eduardoi* (Diptera: Culicidae) in an irrigated area of Patagonia, Chubut Province, Argentina. Mem Inst Oswaldo Cruz. 2007;102:237–9.

Burroni N, Loetti V, Marinone MC, Freire MG, Schweigmann N. Larval habitat of *Ochlerotatus albifasciatus* (Diptera: Culicidae) in the southern edge of the Americas, Tierra del Fuego Island. Open J Anim Sci. 2013;3:5–10.

Grech M, Visintin A, Laurito M, Estallo E, Lorenzo P, Roccia I, et al. New records of mosquito species (Diptera: Culicidae) from Neuquén and La Rioja provinces, Argentina. Rev Saúde Pública. 2012;46:387–9.

Laurito M, Almirón WR, Rossi GC. Description of the immature stages of *Culex* (*Culex*) *ameliae* Casal and *Culex* (*Culex*) *articularis* Philippi (Diptera: Culicidae). Zootaxa. 2011;2778:58–68.

Marinone MC. Nuevas localidades y observaciones sobre la presencia de *Ochlerotatus albifasciatus* (Diptera: Culicidae) en la Patagonia Austral. Rev Soc Entomol Argent. 2002;2:46–7.

Muzón J, Spinelli GR, Pessacq P, von Ellenriedeer N, Estevez AL, Díaz F, et al. Insectos acuáticos de la meseta del Somuncurá, Patagonia, Argentina. Inventario preliminar. Rev Soc Entomol Argent. 2005;64:47–67.

Muzón J, Spinelli GR, Rossi GC, Marino PI, Díaz F, Melo MC. Nuevas citas de insectos acuáticos para la Meseta de Somuncurá, Patagonia, Argentina. Rev Soc Entomol Argent. 2010;69:111–6.

Rossi GC, Laurito M, Almirón WR. Morphological description of the pupa and redescription of the adults and larva of *Culex* (*Culex*) *apicinus* Philippi (Diptera: Culicidae). Zootaxa. 2008;1941:31–42.

Rossi G, Vezzani D. An update of mosquitoes of Argentine Patagonia with new distribution records. J Am Mosq Control Assoc. 2011;27:93–8.

Rossi GC. Annotated checklist, distribution, and taxonomic bibliography of the mosquitoes (Insecta: Diptera: Culicidae) of Argentina. Check List. 2015;11:1712.
