# Supplementary material for: Mosquito (Diptera: Culicidae) larval ecology in natural habitats in the cold temperate Patagonia region of Argentina
Source: Parasit Vectors. 2019 May 7;12:214. doi: 10.1186/s13071-019-3459-y (PMC6505294; doi:10.1186/s13071-019-3459-y)
Supplement: Supplementary file 2 — Additional file 2: Table S2. Description of mosquito larval habitats employed in this study. Geographical coordinates, altitude (masl) and ecoregions are provided. Patagonian provinces: Neuquén (NQ), Río Negro (RN), Chubut (CH), Santa Cruz (SC) and Tierra del Fuego (TF). [file 13071_2019_3459_MOESM2_ESM.docx]

**Additional file 2: Supplementary Table 2.**

| **Site** | **Latitude** | **Longitude** | | **Altitude** | | **Eco-region** | | **Habitat description** | | |
| --- | --- | --- | --- | --- | --- | --- | --- | --- | --- | --- |
| NQ1 | 38°56′36″S | 69°8′46.2″W | | 533 | | Monte | | Animal footprint, clear water, without aquatic vegetation, sunny. | | |
| NQ2 | 38°57′37″S | 69°8′46.2″W | | 533 | | Monte | | Natural temporary pool, clear water, without aquatic vegetation, sunny | | |
| NQ3 | 38°12′19.6″S | 70°36′34.6″W | | 890 | | Steppe | | Natural temporary ditch, clear water, with aquatic vegetation, shade | | |
| NQ4 | 39°28′47.4″S | 70°56′45.9″W | | 814 | | Steppe | | Natural temporary pool, clear water, with aquatic vegetation, shade | | |
| NQ5 | 39°30′14.2″S | 70°57′54.6″W | | 824 | | Steppe | | Natural temporary pool, clear water, with aquatic vegetation, sunny | | |
| NQ6 | 39°41′2″S | 70°58′30.9″W | | 1112 | | Steppe | | Natural temporary pond, clear water, with aquatic vegetation, sunny | | |
| NQ7 | 39°46′52.4″S | 71°2′15.2″W | | 772 | | Forest | | Natural temporary pool, turbid water, with aquatic vegetation, shade | | |
| NQ8 | 40°8′0.7″S | 71°22′15.9″W | | 1163 | | Forest | | Natural temporary pond, clear water, with aquatic vegetation, sunny | | |
| RN1 | 39°25′38″S | 65°40′23.9″W | | 106 | | Monte | | Natural temporary pool, turbid water, with aquatic vegetation, shade | | |
| RN2 | 39°25′37.3″S | 65°40′25.5″W | | 238 | | Monte | | Artificial temporary ditch, turbid water, with aquatic vegetation, sunny | | |
| CH1 | 42°39′8.6″S | 70°6′9.7″W | | 441 | | Steppe | | Natural temporary pool, turbid water, with aquatic vegetation, sunny | | |
| CH2 | 42°30′16.9″S | 71°31′12.3″W | | 530 | | Forest | | Natural temporary ditch, clear water, with aquatic vegetation, sunny | | |
| CH3 | 43°8′26″S | 71°38′1.3″W | | 378 | | Forest | | Natural temporary pool, clear water, with aquatic vegetation, shade | | |
| CH4 | 43°8′27.3″S | 71°38′1.7″W | | 377 | | Forest | | Natural temporary pool, clear water, with aquatic vegetation, sunny | | |
| CH5 | 43°8′59.8″S | 71°8′30.1″W | | 373 | | Steppe | | Rock pool, clear water, with aquatic vegetation, shade | | |
| CH6 | 43°17′38.2″S | 65°30′41.6″W | | 14 | | Monte | | Natural temporary pool, turbid water, with aquatic vegetation, shade | | |
| CH7 | 42°2′29.4″S | 71°32′54.4″W | | 319 | | Forest | | Artificial temporary pond, clear water, without aquatic vegetation, shade | | |
| CH8 | 43°7′25.5″S | 71°26′44.7″W | | 350 | | Steppe | | Natural temporary pool, clear water, with aquatic vegetation, sunny | | |
| CH9 | 44°9′23.3″S | 71°26′50.7″W | | 723 | | Steppe | | Natural temporary pool, turbid water, with aquatic vegetation, sunny | | |
| CH10 | 45°36′13.8″S | 69°3′0.1″W | | 126 | | Steppe | | Natural temporary pool, clear water, with aquatic vegetation, sunny | | |
| SC1 | 46°36′51.2″S | 71°38′11.8″W | | 314 | | Steppe | | Natural temporary pool, clear water, with aquatic vegetation, sunny | | |
| SC2 | 49°4′42.1″S | 72°53′18.4″W | | 507 | | Forest | | Animal footprint, clear water, with aquatic vegetation, sunny | | |
| SC3 | 50°28′18.3″S | 73°01′49.9″W | | 263 | | Forest | | Natural temporary pond, clear water, with aquatic vegetation, sunny | | |
| TF1 | 54°24′6.8″S | 77°14′25.15″W | | 33 | | Forest | | Natural temporary ditch, clear water, with aquatic vegetation, sunny | | |
| TF2 | 54°35′42.7″S | | 67°22′12.4″W | | 26 | | Forest | | Natural temporary pool, turbid water, with aquatic vegetation, sunny |  |
| TF3 | 54°21′40.2″S | | 67°39′11.7″W | | 82 | | Forest | | Natural temporary pool, turbid water, with aquatic vegetation, sunny |  |
