# Supplementary material for: Mosquito (Diptera: Culicidae) larval ecology in natural habitats in the cold temperate Patagonia region of Argentina
Source: Parasit Vectors. 2019 May 7;12:214. doi: 10.1186/s13071-019-3459-y (PMC6505294; doi:10.1186/s13071-019-3459-y)
Supplement: Supplementary file 3 — Additional file 3: Table S3. Spearman rank correlations matrix. Spearman correlations between all environmental variables measured in the 26 mosquito larval habitats, climate variables extracted from WorldClim v.2 and MODIS vegetation indices. Variables: pH; WT, water temperature; conductivity; TDS, total dissolved solid; DO, dissolved oxygen; salinity; alkalinity; APC, aquatic plant cover; PRS, soluble reactive phosphorous; NO3-+NO2+, nitrate+nitrite; NH4+, ammonia; WD, water depth; area; Air T, in situ air temperature; Hydric B, hydric balance; Wind S, wind speed; temperature; Solar R, solar radiation; precipitation; Min CM, minimum temperature of coldest month; Max WM, maximum temperature of warmest month; Pre WM, precipitation of wettest month; Pre DM, precipitation of driest month; NDVI; EVI; latitude and longitude. Correlation coefficients higher than 0.5 are highlighted in bold. [file 13071_2019_3459_MOESM3_ESM.docx]

**Additional file 3: Supplementary Table 3.**

|  | pH | WT | Conductivity | TDS | DO | Salinity | Alkalinity | APC | PRS | NO_3_^-^+NO_2_^-^ | NH_4_^+^ | WD | Area | Air T | Hydric B | Wind S | Temperature | Solar R | Precipitation | Min CM | Max WM | Pre WM | Pre DM | NDVI | EVI | Latitude | Longitude |
| --- | --- | --- | --- | --- | --- | --- | --- | --- | --- | --- | --- | --- | --- | --- | --- | --- | --- | --- | --- | --- | --- | --- | --- | --- | --- | --- | --- |
| pH | **1** | - | - | - | - | - | - | - | - | - | - | - | - | - | - | - | - | - | - | - | - | - | - | - | - | - | - |
| WT | **0.6** | **1** | - | - | - | - | - | - | - | - | - | - | - | - | - | - | - | - | - | - | - | - | - | - | - | - | - |
| Conductivity | **0.6** | 0.3 | **1** | - | - | - | - | - | - | - | - | - | - | - | - | - | - | - | - | - | - | - | - | - | - | - | - |
| TDS | **0.6** | 0.3 | **1** | **1** | - | - | - | - | - | - | - | - | - | - | - | - | - | - | - | - | - | - | - | - | - | - | - |
| DO | 0.2 | -0.2 | 0.2 | 0.2 | **1** | - | - | - | - | - | - | - | - | - | - | - | - | - | - | - | - | - | - | - | - | - | - |
| Salinity | **0.5** | 0.3 | **0.9** | **0.9** | 0.3 | **1** | - | - | - | - | - | - | - | - | - | - | - | - | - | - | - | - | - | - | - | - | - |
| Alkalinity | **0.8** | **0.6** | **0.8** | **0.8** | 0.1 | **0.7** | **1** | - | - | - | - | - | - | - | - | - | - | - | - | - | - | - | - | - | - | - | - |
| APC | -0.2 | -0.1 | -0.2 | -0.3 | 0.1 | -0.2 | -0.2 | **1** | - | - | - | - | - | - | - | - | - | - | - | - | - | - | - | - | - | - | - |
| PRS | 0.4 | 0.3 | **0.5** | **0.5** | 0.1 | **0.5** | **0.5** | -0.1 | **1** | - | - | - | - | - | - | - | - | - | - | - | - | - | - | - | - | - | - |
| NO_3_^-^+NO_2_^-^ | -0.03 | 0.02 | -0.1 | -0.1 | -0.1 | -0.2 | 0.01 | 0.01 | 0.1 | **1** | - | - | - | - | - | - | - | - | - | - | - | - | - | - | - | - | - |
| NH_4_^+^ | 0.07 | 0.1 | 0.3 | 0.3 | -0.1 | 0.4 | 0.2 | 0.2 | 0.3 | 0.4 | **1** | - | - | - | - | - | - | - | - | - | - | - | - | - | - | - | - |
| WD | 0.03 | 0.2 | -0.2 | -0.2 | -0.2 | -0.3 | -0.1 | 0.2 | **-0.5** | -0.2 | -0.04 | **1** | - | - | - | - | - | - | - | - | - | - | - | - | - | - | - |
| Area | **-0.5** | 0.01 | **-0.5** | **-0.6** | -0.3 | **-0.6** | -0.3 | **0.5** | -0.2 | 0.2 | 0.1 | 0.3 | **1** | - | - | - | - | - | - | - | - | - | - | - | - | - | - |
| Air T | 0.3 | **0.6** | 0.3 | 0.3 | -0.2 | 0.2 | **0.6** | -0.4 | 0.3 | 0.1 | -0.1 | 0.01 | -0.0003 | **1** | - | - | - | - | - | - | - | - | - | - | - | - | - |
| Hydric B | **-0.5** | -0.2 | **-0.7** | **-0.7** | -0.3 | **-0.6** | **-0.6** | 0.03 | **-0.5** | 0.1 | -0.1 | 0.4 | 0.4 | -0.3 | **1** | - | - | - | - | - | - | - | - | - | - | - | - |
| Wind S | -0.4 | **-0.5** | -0.2 | -0.2 | 0.1 | -0.2 | -0.4 | **0.6** | -0.1 | -0.3 | -0.2 | -0.1 | 0.1 | **-0.7** | 0.1 | **1** | - | - | - | - | - | - | - | - | - | - | - |
| Temperature | **0.6** | **0.6** | **0.5** | **0.5** | -0.1 | **0.5** | **0.8** | -0.1 | 0.4 | 0.2 | 0.3 | -0.1 | -0.001 | **0.7** | **-0.5** | **-0.6** | **1** | - | - | - | - | - | - | - | - | - | - |
| Solar R | **0.5** | **0.6** | 0.3 | 0.3 | -0.02 | 0.2 | **0.7** | -0.3 | 0.4 | 0.2 | -0.1 | -0.02 | -0.03 | **0.9** | -0.4 | **-0.7** | **0.8** | **1** | - | - | - | - | - | - | - | - | - |
| Precipitation | -0.4 | -0.2 | **-0.6** | **-0.6** | -0.4 | **-0.6** | **-0.5** | 0.1 | **-0.6** | 0.2 | -0.1 | **0.5** | 0.4 | -0.2 | **0.9** | -0.01 | -0.4 | -0.3 | **1** | - | - | - | - | - | - | - | - |
| Min CM | 0.2 | 0.2 | **0.5** | **0.5** | 0.03 | **0.5** | 0.4 | 0.2 | 0.3 | -0.1 | **0.5** | -0.2 | 0.1 | 0.1 | -0.4 | -0.1 | 0.5 | 0.1 | -0.4 | **1** | - | - | - | - | - | - | - |
| Max WM | **0.6** | **0.6** | **0.5** | **0.5** | -0.03 | 0.4 | **0.8** | -0.2 | **0.5** | 0.2 | 0.2 | -0.2 | -0.04 | **0.8** | **-0.6** | **-0.6** | **0.9** | **0.9** | **-0.5** | 0.3 | **1** | - | - | - | - | - | - |
| Pre WM | -0.2 | -0.04 | **-0.5** | **-0.5** | -0.4 | **-0.5** | -0.4 | -0.01 | **-0.6** | 0.3 | -0.1 | **0.6** | 0.3 | -0.1 | **0.9** | -0.1 | -0.3 | -0.2 | **1** | **-0.5** | -0.4 | **1** | - | - | - | - | - |
| Pre DM | **-0.5** | -0.3 | **-0.5** | **-0.5** | -0.3 | **-0.5** | **-0.6** | 0.1 | **-0.7** | 0.1 | 0.02 | 0.4 | 0.4 | -0.4 | **0.8** | 0.1 | **-0.5** | **-0.5** | **0.9** | -0.1 | **-0.6** | **0.7** | **1** | - | - | - | - |
| NDVI | **-0.6** | -0.2 | -0.4 | -0.4 | **-0.5** | -0.4 | **-0.6** | 0.3 | -0.4 | 0.03 | 0.3 | 0.3 | 0.4 | -0.4 | **0.7** | 0.2 | -0.4 | **-0.6** | **0.6** | 0.1 | **-0.6** | **0.5** | **0.7** | **1** | - | - | - |
| EVI | **-0.5** | -0.1 | -0.4 | -0.3 | **-0.6** | -0.3 | -0.4 | 0.3 | -0.2 | 0.04 | 0.4 | 0.2 | **0.6** | -0.3 | **0.5** | 0.2 | -0.2 | -0.4 | **0.5** | 0.2 | -0.4 | 0.3 | **0.5** | **0.9** | **1** | - | - |
| Latitude | **-0.5** | **-0.7** | -0.2 | -0.2 | 0.02 | -0.2 | **-0.5** | 0.4 | -0.2 | -0.3 | 0.1 | -0.03 | 0.03 | **-0.9** | 0.2 | **0.9** | **-0.7** | **-0.9** | 0.1 | -0.02 | **-0.8** | -0.03 | 0.4 | **0.5** | 0.4 | **1** | - |
| Longitude | -0.1 | -0.1 | **-0.6** | **-0.6** | -0.2 | **-0.5** | -0.3 | 0.1 | **-0.5** | -0.2 | -0.4 | 0.4 | 0.2 | -0.3 | **0.7** | 0.2 | -0.3 | -0.3 | **0.7** | -0.4 | -0.4 | **0.6** | **0.6** | 0.3 | 0.2 | 0.2 | **1** |
