# Supplementary material for: Mosquito (Diptera: Culicidae) larval ecology in natural habitats in the cold temperate Patagonia region of Argentina
Source: Parasit Vectors. 2019 May 7;12:214. doi: 10.1186/s13071-019-3459-y (PMC6505294; doi:10.1186/s13071-019-3459-y)
Supplement: Supplementary file 4 — Additional file 4: Table S4. Species richness estimators. Species richness estimators (± standard error) and number of observed species for the total of the 26 mosquito larval habitats, and for each site. Chao; Jack1: first order jackknife; Jack2: second order jackknife and Boot: bootstrap. [file 13071_2019_3459_MOESM4_ESM.docx]

**Additional file 4: Supplementary Table 4.**

| **Sites** | **Observed species** | **Chao ± EE** | **Jack1 ± EE** | **Jack2** | **Boot ± EE** |
| --- | --- | --- | --- | --- | --- |
| 26 | 7 | 11.3±6.9 | 9.9±1.7 | 11.8 | 8.2±0.9 |
| CH1 | 1 | 1±0 | - | - | - |
| CH2 | 1 | 1±0 | - | - | - |
| CH3 | 1 | 1±0 | - | - | - |
| CH4 | 1 | 1±0 | - | - | - |
| CH5 | 2 | 2±0 | - | - | - |
| CH6 | 2 | 2±0.4 | - | - | - |
| CH7 | 1 | 1±0 | - | - | - |
| CH8 | 1 | 1±0 | - | - | - |
| CH9 | 1 | 1±0 | - | - | - |
| CH10 | 1 | 1±0 | - | - | - |
| RN1 | 1 | 1±0 | - | - | - |
| RN2 | 1 | 1±0 | - | - | - |
| NQ1 | 2 | 2±0 | - | - | - |
| NQ2 | 1 | 1±0 | - | - | - |
| NQ3 | 1 | 1±0 | - | - | - |
| NQ4 | 1 | 1±0 | - | - | - |
| NQ5 | 1 | 1±0 | - | - | - |
| NQ6 | 1 | 1±0 | - | - | - |
| NQ7 | 1 | 1±0 | - | - | - |
| NQ8 | 1 | 1±0 | - | - | - |
| SC1 | 1 | 1±0 | - | - | - |
| SC2 | 1 | 1±0 | - | - | - |
| SC3 | 1 | 1±0 | - | - | - |
| TF1 | 1 | 1±0 | - | - | - |
| TF2 | 1 | 1±0 | - | - | - |
| TF3 | 1 | 1±0 | - | - | - |
