# Supplementary figures and images for: Mosquito (Diptera: Culicidae) larval ecology in natural habitats in the cold temperate Patagonia region of Argentina
Source: Parasit Vectors. 2019 May 7;12:214. doi: 10.1186/s13071-019-3459-y (PMC6505294; doi:10.1186/s13071-019-3459-y)

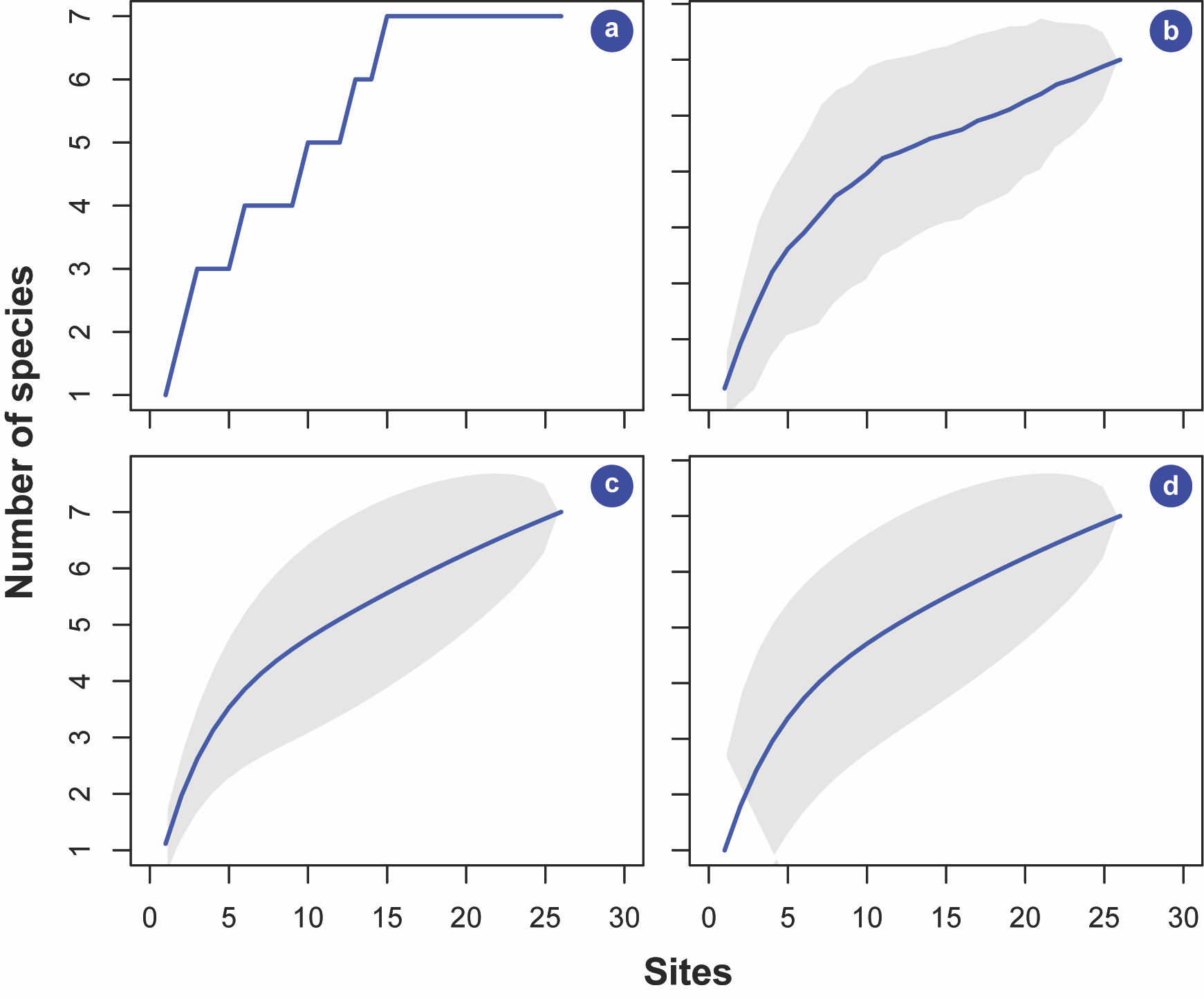

Supplement: Supplementary file 5 — Additional file 5: Figure S1.Species accumulation curves. Species accumulation curves for the 26 mosquito larval habitats, using the species accumulation methods: collector (a), random (b), exact (c) and coleman (d). [file 13071_2019_3459_MOESM5_ESM.tiff]
